# Recent and dynamic transposable elements contribute to genomic divergence under asexuality

**DOI:** 10.1186/s12864-016-3234-9

**Published:** 2016-11-07

**Authors:** Julie Ferreira de Carvalho, Victor de Jager, Thomas P. van Gurp, Niels C. A. M. Wagemaker, Koen J. F. Verhoeven

**Affiliations:** 1 Department of Terrestrial Ecology, Netherlands Institute of Ecology (NIOO-KNAW), Droevendaalsesteeg 10, 6708 PB Wageningen, The Netherlands; 2 Bioinformatic Support Group, Netherlands Institute of Ecology (NIOO-KNAW), Droevendaalsesteeg 10, 6708 PB Wageningen, The Netherlands; 3 Experimental Plant Ecology, Radboud University Nijmegen, Heyendaalseweg 135, 6525 AJ Nijmegen, The Netherlands

**Keywords:** Transposable elements, Methylation, Asexuality, Apomixis, Genome evolution, *Taraxacum officinale*, Dandelion

## Abstract

**Background:**

Transposable elements (TEs) are mobile pieces of genetic information with high mutagenic potential for the host genome. Transposition is often neutral or deleterious but may also generate potentially adaptive genetic variation. This additional source of variation could be especially relevant in non-recombining species reproducing asexually. However, evidence is lacking to determine the relevance of TEs in plant asexual genome evolution and their associated effects. Here, we characterize the repetitive fraction of the genome of the common dandelion, *Taraxacum officinale* and compare it between five accessions from the same apomictic lineage. The main objective of this study is to evaluate the extent of within-lineage divergence attributed to TE content and activity. We examined the repetitive genomic contribution, diversity, transcription and methylation changes to characterize accession-specific TEs.

**Results:**

Using low-coverage genomic sequencing, we report a highly heterogeneous TE compartment in the triploid apomict *T. officinale* representing up to 38.6 % of the homoploid genome. The repetitive compartment is dominated by LTR retrotransposon families accompanied by few non-LTR retrotransposons and DNA transposons. Up to half of the repeat clusters are biased towards very high read identity, indicating recent and potentially ongoing activity of these TE families. Interestingly, the five accessions are divided into two main clades based on their TE composition. Clade 2 is more dynamic than clade 1 with higher abundance of *Gypsy Chromovirus* sequences and transposons. Furthermore, a few low-abundant genomic TE clusters exhibit high level of transcription in two of the accessions analysed. Using reduced representation bisulfite sequencing, we detected 18.9 % of loci differentially methylated, of which 25.4 and 40.7 % are annotated as TEs or functional genes, respectively. Additionally, we show clear evidence for accession-specific TE families that are differentially transcribed and differentially methylated within the apomictic lineage, including one *Copia Ale II* LTR element and a *PIF-Harbinger* DNA transposon.

**Conclusion:**

We report here a very young and dynamic repetitive compartment that enhances divergence within one asexual lineage of *T. officinale*. We speculate that accession-specific TE families that are both transcriptionally and epigenetically variable are more prone to trigger changes in expression on nearby coding sequences. These findings emphasize the potential of TE-induced mutations on functional genes during asexual genome evolution.

**Electronic supplementary material:**

The online version of this article (doi:10.1186/s12864-016-3234-9) contains supplementary material, which is available to authorized users.

## Background

Transposable elements (TEs) are mobile pieces of DNA sequences. They are classified according to their mode of replication. Class I retrotransposon elements copy and paste through RNA sequences whereas Class II DNA transposons excise and insert into a new location as DNA molecules [1]. Each class is subdivided into super-families and families based on their conserved coding domains. In higher plants, the repetitive fraction can represent more than 80 % of genomes [2, 3]. The dynamic nature of TEs triggers important effects on genetic variability and genomic architecture [4]. They can create mutations, insertion breaks and promote chromosomal aberrations. Also, TEs can alter host gene expression via changes in promoter regions or antisense regulation, or through epigenetic repression affecting targeted TE and nearby genes [5]. Epigenetic marks and more specifically DNA methylation regulation can be dynamic and de-suppression has been shown following environmental stresses experienced by the host [6]. As a result, TEs can be reactivated contributing to rapid intraspecific variation in natural system [7, 8]. Mobilized TEs may have neutral or deleterious effects but they can also facilitate evolution, supporting the production and maintenance of genetic variation [9].

Theoretical and empirical work suggests that the host reproduction mode has an important effect on TE content. The evolution and dynamics of TEs reflect the combined effects of selection and TE transmission [10, 11]. In asexual plant species, within-lineage transmission of genetic information is expected to limit TE proliferation over time due to the absence of meiosis and the inability of TEs to transfer to a different genetic background [12]. However, asexual plant lineages usually derive from sexual ancestors, thus inheriting the TE load from their sexual relatives. Following transition to asexuality, due to less efficient selection against deleterious mutations, original TE load might initially increase due to a Muller’s ratchet-like process [10, 13]. Additionally, transition from a sexual to an asexual mating system is often coupled to changes in ploidy level and/or an intraspecific hybridization event. Following such genomic events, epigenetic mechanisms controlling TE activity can be disrupted which can lead to a rapid increase in TE copy number and/or activity [14]. During this process, a subset of elements might escape epigenetic control. Over evolutionary time, selection against asexual lineages with high TE proliferation rates is thought to result in the selective persistence of lineages where most of TEs are efficiently controlled and silenced, not disturbing the genomic architecture of the host genome. Thus, the dynamics of TE accumulation and extinction appears as a specific and dynamic evolutionary process within each asexual lineage.

Young and re-mobilized TEs appear to trigger the most effects on host silencing mechanism and on genomic disruption compared to older elements [15]. Recently, a set of young, active and recently inserted TEs have been found occurring in the extremely repetitive maize genome [16]. These elements, mostly low-copy number LTR elements, are highly expressed and targeted by siRNAs, whereas ancient and “mostly-dead” elements are under strong transposition control with high copy number coupled with low-expression [16]. Age of insertion, integrity of coding domains and efficiency of the host genome regulation are highly TE family specific [16–18]. These effects could lead to extreme TE divergence within asexual lineages.

Effects of TE-associated mutations are expected to be neutral or deleterious according to the genomic context of insertion, and asexual lineages with high TE load are expected to be selected against. However, positive TE-associated mutations inducing beneficial phenotypes could be retained while mild deleterious mutations are buffered by polyploidy. Increased genetic diversity for instance during brief periods of stress can have short-term adaptive benefits in asexuals [19]. Indeed, epigenetic TE silencing mechanisms can be altered, enhancing mutation rates and possibly providing a fine-tuning mechanism to respond to unstable environmental conditions [20, 21]. Furthermore, recently mobilized TEs could provide an additional source of genetic diversity for asexual lineages that may be constrained in their adaptive potential by limited genetic variation [22].

Widespread apomictic lineages of the common dandelion, *Taraxacum officinale* provide an excellent system to evaluate TE dynamics under asexual reproduction. Derived from sexual diploid ancestors in South-Central Europe [23, 24], dandelions from Northern Europe are usually triploid apomicts (2n = 3x = 24). Backcrosses between apomictic pollen donors and sexual mother plants occur in the hybridization zone, leading to the creation of new apomictic lineages [24]. This process has led to numerous widespread apomictic lineages [25]. We observed previously that active TE and TE-related genes are an important component of transcriptomic divergence within such apomictic lineages [26]. We hypothesized that recent and dynamic TEs are triggering most of the genomic differences observed within apomictic lineages.

Here, we characterize the repeat fraction in the *T. officinale* genome and pinpoint differences between plants from a single widespread apomictic lineage. Specifically, we aim to (1) characterize the repeat fraction of *T. officinale* genome; (2) use genomic and transcriptomic information to reveal past and ongoing activity of specific TE families; and (3) unravel differences in TE activity and methylation regulation between natural accessions of a single widespread apomictic lineage. Together, these analyses provide unique insight in the importance of TEs for driving genomic divergence within asexual plant lineages. We used whole genome sequencing and a clustering-based analysis pipeline to annotate the repetitive compartment of *T. officinale*. We report a highly heterogeneous low-abundant and dynamic TE compartment in the genome. Furthermore, we characterized a high proportion of recent and transcribed TE families contributing to genomic divergence under asexuality. We discuss the possible implications of such findings in our understanding of asexual genome evolution.

## Methods

### Plant material and DNA extractions

We selected five natural accessions of the apomictic *T. officinale* lineage Macranthoides to document and compare their repetitive genomic fractions. Macranthoides is a geographically widespread apomictic dandelion lineage, also referred to as a microspecies, that can be identified by expert taxonomist and whose unique clonal nature was confirmed by identical multi-locus microsatellite genotypes [25]. The same five accessions were previously screened for heritable divergence in gene expression using RNA-Seq [26]. The accessions were identified by Taraxacum taxonomists and sampled in Germany and Czech Republic from 3 different field locations (maximum distance 300 km) during spring 2012 (Additional file 1). Seeds were propagated for two generations in a common greenhouse environment. For each generation, seeds were surface-sterilized with a 0.5 % Sodium Hypochlorite solution and were germinated on agar (0.8 %) in petri dishes for 10 days in a climate chamber (10 h dark/14 h light, 15/20 °C). Then, seedlings were transplanted to individual pots (80 % potting soil with 20 % pumice) and grown in the greenhouse until leaf collection. For subsequent DNA extractions, 10 discs from two young leaves of the same individual were punched, pooled in one tube and directly frozen in liquid nitrogen. Samples were stored at −80 °C until DNA extractions.

Genomic DNA was isolated using NucleoSpin Plant II (Macherey-Nagel) columns following the manufacturer’s protocol with modifications as follows: leaf tissue was finely ground using liquid nitrogen before adding PL1 cell lysis buffer and RNase A solution. Lysate was incubated at 65 °C for 30 min and centrifuged for 2 min at full speed. Only supernatant was loaded onto the column. Quality and concentration were checked on a NanoDrop 1000 spectrophotometer.

### Illumina sequencing

Sequencing of the five accession total genomic DNA was conducted by the CAT-AgroFood sequencing facility (Wageningen University, NL). Libraries with average insert size of 500 bp were prepared and pooled for subsequent 125 bp paired-end sequencing using half a lane of an Illumina HiSeq 2500 system. Genomic reads were analysed and trimmed for adapters and low-quality sequences using FastQC quality control. Cleaned sequencing output for each accession led to 41.4, 32.8, 55.0, 59.4 and 51.8 million reads for accessions 8, 11, 3, 12 and 13 respectively. From each accession a random sample of 5 million reads (125 bp length) was drawn from the quality-filtered sequencing output for subsequent downstream TE analysis that is comparable between the five accessions, representing 24 % of *T. officinale* triploid genome.

### *De novo* identification of repetitive sequence families in *Taraxacum*

The RepeatExplorer pipeline via a Galaxy interface was applied to our datasets in order to cluster and assemble consensus sequences (contigs) of highly repeated fractions of the genome [27]. The five accessions were analysed separately. Read clustering criteria included 55 % minimum overlap and 90 % minimum similarity in the overlapping region. All clusters containing at least 0.2 % of the input reads were examined manually to identify clusters that required merging following recommendations and parameter settings from Kelly et al. [28]. Clusters were annotated according to similarity searches against the RepeatMasker Viridiplantae Database [29], the Conserved Domain Database [30] and GenBank non-redundant genomic database [31]. Chloroplast sequences were also clustered and excluded from subsequent analyses. After merging same-annotation clusters, all clusters representing >0.2 % of the input reads were explored manually to check for consistency in repeat annotation. This subset of high-abundant repeats was used for all subsequent analyses. To calculate the different repeat fraction sizes, total read lengths of same-annotation clusters representing similar TE clades, sub-clades, superfamilies and when possible families, were summed up [1]. Genome proportions were calculated based on the average monoploid genome size i.e. 865 Mb as calculated by Záveský et al. [32]. Differences in relative abundance of TE classes between accessions, as estimated by read counts, were tested using the likelihood ratio Chi-square (SAS 9.2, The SAS Institute, Carey NC).

### Intra-family heterogeneity of repeats

To determine the level of mutational divergence between repeat copies within each cluster, we conducted a BLASTn all against all reads following recommendations by Kelly et al. [28]. Self-hit blast results were removed from the output files. For each cluster, a histogram of the pairwise percentage identity scores (between 0.9 and 1.0) was constructed using R [33]. Clusters with a bias towards high read similarity reflect sequence conservation and possible recent proliferation; whereas clusters for which the majority of reads have lower similarity scores reflect older families that exhibit mutational divergence. To automatically analyse patterns in the similarity histograms, two regression models (linear: Y = a + bX; and quadratic: Y = a + bX + cX^2^) were fitted to the histogram of each cluster using a custom R script. Regression coefficients were extracted from the best-fitting model based on the Bayesian Information Criterion [34]. Based on these regression coefficients, we classified individual clusters into six categories: 1) positive linear regression (linear model has lowest BIC and b > 0.001), 2) absence of linear relation (linear model has lowest BIC and −0.001 < b < 0.001), 3) negative linear regression (linear model has lowest BIC and b < −0.001), 4) positive quadratic function (quadratic model has lowest BIC and curve opens upward, b^2^ > 0), 5) negative quadratic function (quadratic model has lowest BIC, curve opens downward b^2^ < 0 and optimum is below 99 % pairwise identity) and 6) negative quadratic function with high optimum (quadratic model has lowest BIC, curve opens downward b^2^ < 0 and optimum is above 99 % pairwise identity). Based on this classification, we identified TE clusters showing high sequence similarity of copies as a proxy for recent activity and proliferation and we distinguished them from low similarity clusters representing older copies accumulating mutations [28, 35]. We interpreted categories 1, 4 and 6 as evidence for young and dynamic TEs, and 2, 3, 5 as older and well-silenced TEs. This regression approach is a simple heuristic method to distinguish between these categories and is not intended to detect statistically significant differences between clusters.

### TE family genomic content and level of transcription

From a previous study, we developed Illumina transcriptomic resources for these same *T. officinale* Macranthoides accessions [26]. For each accession, contigs assembled through the RepeatExplorer pipeline were used as a new TE database to which genomic and transcriptomic Illumina reads were mapped. This analysis enabled exploration of the most abundant TE family transcription activity at the cluster level. Both genomic and transcriptomic single reads were aligned and read abundance estimated per cluster using the Trinity pipeline, including Bowtie2 and RSEM methods [36–38]. A quality score was given for each alignment and the best one was kept for abundance estimation. Log ratios of FPKM normalized values (as described by [39]) were used to quantify transcription for each cluster. For each accession, we used simple linear regression to test for a relationship across all clusters between genomic abundance and expression.

### Methylation profiles of the *T. officinale* accessions

To assess differential methylation levels in TE sequences among accessions, we used a recently developed reduced representation bisulfite sequencing (RRBS) protocol that does not require a reference genome [40]. Individuals originating from the same mother plants as the ones used for genomic sequencing were grown in a common greenhouse environment. DNA was extracted from leaf tissue as described above. For each accession, following van Gurp et al. [40], reduced genomic libraries were generated by *PstI* digestion, size selection and bisulfite treatment to convert unmethylated (but not methylated) cytosines before being pooled and sequenced on ¼ lane of Illumina Hi-Seq 2500.

Bioinformatics analysis for methylation calling followed van Gurp et al. [40]. Briefly, in-line barcodes present in the reads were stripped and a sample-specific read group tag was added to the read name. Overlapping paired-end reads were merged using PEAR [41]. Merged and non-merged reads were then mapped using BWA-Meth [42] to the pseudo-reference sequence of the *T. officinale* genome constructed previously by van Gurp et al. [40]. The first four adapter-derived nucleotides were clipped from the merged and non-merged reads. Watson and Crick specific bam files representing both DNA strands were created, identifying methylation polymorphisms as positions where (a proportion of) cytosines were converted, but guanines on the opposite strand were unmodified. Both Watson and Crick bam files were processed through Freebayes to identify polymorphic variants (with following parameters -F 0 -E 1 -C 0 -G 0 --haplotype-length 1 --report-all-haplotype-alleles --report-monomorphic --report-genotype-likelihood-max --haplotype-length 1) [43]. Combining variant calls from both Watson and Crick strands allowed for distinguishing genetic from epigenetic variation [44]. Resulting variants were merged into two files: one for single methylation polymorphisms, the other for genetic polymorphisms using custom-made scripts freely available in github.

The methylation output file included information on location and level of methylation for each cytosine position called. Methylation proportions were calculated as number of reads with methylated position divided by total number of reads matching that same position. Following analyses were based on a subset of highly confident positions called in all 5 accessions with a minimum of 50x coverage. Out of the 40,456 cytosines in the RRBS data, 4733 were found in CG context. Those positions were occurring on 623 RRBS loci. Because methylation in CG context is highly bimodal (Additional file 2) with usually very high or very low methylation proportion [45] we collapsed CG quantitative methylation calls in three qualitative levels: low methylation (<25 %), intermediate methylation (25–75 %) and high methylation (>75 %). We defined a “true” methylation polymorphism (differentially methylated position or DMP) if the five accessions showed variation ranging from low to high methylation levels. However, a larger number of positions showed variation between accessions from low to intermediate methylation or from intermediate to high levels of methylation and are therefore included in the analysis. As multiple DMPs can be clustered on the same locus, we also calculated the average level of methylation across all CG positions within each RRBS locus, to identify Differentially Methylated Regions (DMRs). DMRs were called using the same qualitative thresholds as for DMPs. Using BLAST, homologous sequences corresponding to all filtered loci and the DMR subset were retrieved from the genomic TE database, reference transcriptome and subsequent RNA-Seq data [26]. We were thus able to annotate each loci as TE, functional transcripts or unknown. For each locus annotated as TE, transcript level was retrieved in all accessions.

## Results

### The repetitive landscape of *T. officinale* apomictic genomes

**Fig. 1:**
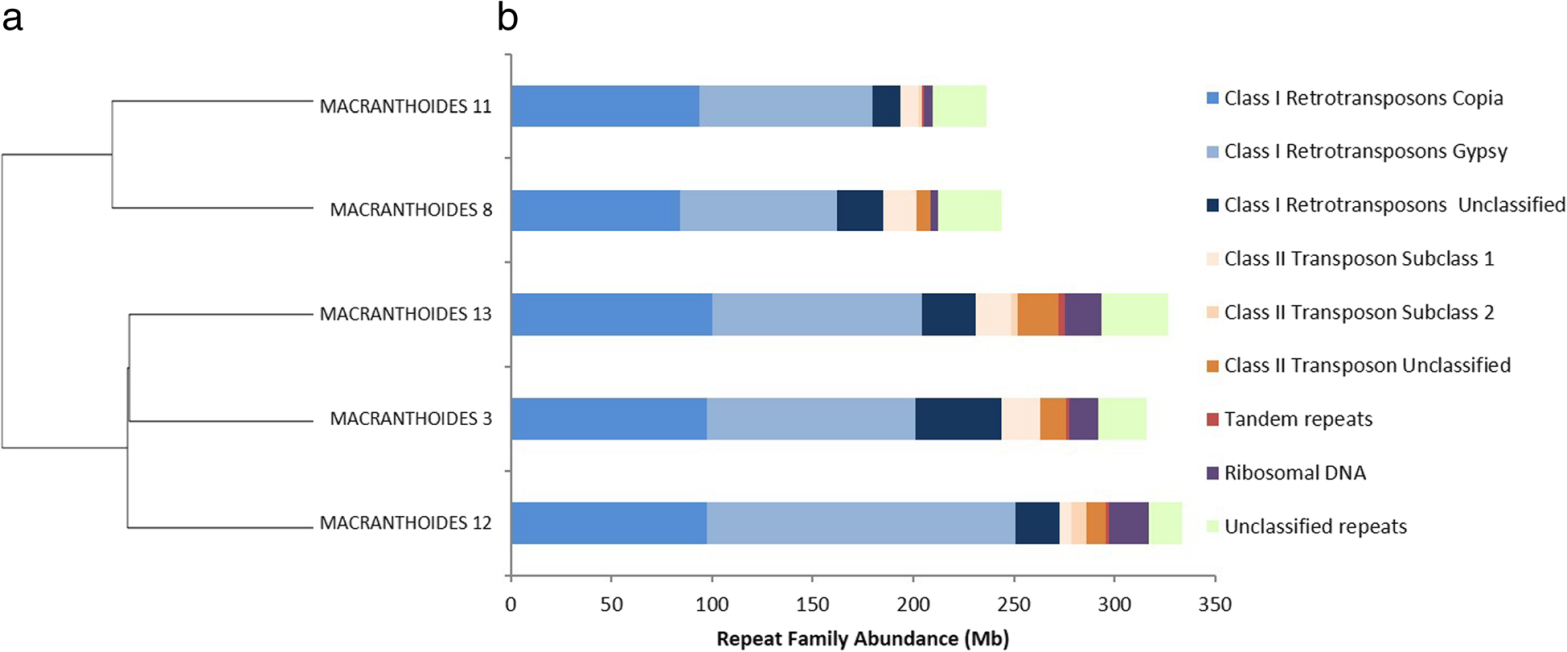
Genomic abundance of TE superfamilies in five accessions of one apomictic lineage of Taraxacum officinale. **a** Clustering from previous SNP analysis [26], **b** Genomic contributions of the most abundant TE superfamilies, tandem repeat and ribosomal RNA. The x-axis indicates abundance in Mega base pairs for each accession shown on the y-axis

In the five accessions, 96 to 118 clusters were identified, each representing ≥ 0.2 % of input nuclear reads (Table 1). Total lengths and genome proportions for each TE family in the different accessions are shown in Table 1 and Fig. 1. The genomic proportions of highly repetitive DNA varied among accessions, ranging from 27.4 to 38.6 %. Except for *DIRS*, *Penelope* retrotransposons and *TIR* transposons, all superfamilies of TE known in plants were retrieved in the datasets [1]. Most widely distributed were Ty1/*Copia* and Ty3/*Gypsy* retroelements with *Maximus/SIRE* and *Chromovirus* lineages respectively, *hAT* transposons, Tandem repeats and ribosomal DNA. Only few *LINE* elements and other transposon families were found among the five accessions.

**Table 1:** Summary of most abundant transposable element families in all five accessions studied

Annotations	Macranthoides 11				Macranthoides 8				Macranthoides 12				Macranthoides 13				Macranthoides 3			
	Nb clusters	Nb clusters in model 1-4-6	Size (Mbp)	% TE	Nb clusters	Nb clusters in model 1-4-6	Size (Mbp)	% TE	Nb clusters	Nb clusters in model 1-4-6	Size (Mbp)	% TE	Nb clusters	Nb clusters in model 1-4-6	Size (Mbp)	% TE	Nb clusters	Nb clusters in model 1-4-6	Size (Mbp)	% TE
Retrotransposons	76	23	193.42	22.36	74	20	185.16	21.41	88	40	272.43	31.50	78	33	231.15	26.72	88	37	243.70	28.17
LTR Elements Ty1/Copia	38	15	93.59	10.82	37	12	84.05	9.72	36	14	97.63	11.29	36	14	99.99	11.56	38	16	97.32	11.25
LTR Elements Ty3/Gypsy	31	6	85.86	9.93	28	8	78.05	9.02	46	22	152.89	17.67	32	14	104.24	12.05	36	14	103.73	11.99
Unclassified LTR	4	0	8.96	1.04	5	0	13.44	1.55	5	4	18.76	2.17	10	5	26.92	3.11	5	1	11.55	1.33
LINE	1	1	1.77	0.20	0	0	0.00	0.00	0	0	0.00	0.00	0	0	0.00	0.00	2	0	4.52	0.52
ParaRetrovirus (Caulimovirus)	1	1	1.13	0.13	0	0	0.00	0.00	0	0	0.00	0.00	0	0	0.00	0.00	0	0	0.00	0.00
Unclassified retrotransposons	1	0	2.10	0.24	4	0	9.62	1.11	1	0	3.15	0.36	0	0	0.00	0.00	7	6	26.58	3.07
DNA transposons	5	1	10.67	1.23	6	3	23.55	2.72	8	3	23.16	2.68	14	6	41.12	4.75	12	5	32.14	3.72
Unclassified repeats	12	4	26.93	3.11	14	8	31.51	3.64	8	5	16.50	1.91	8	5	32.87	3.80	10	7	24.25	2.80
Tandem repeats	1	1	1.13	0.13	0	0	0.00	0.00	1	1	1.65	0.19	2	2	2.84	0.33	1	1	1.45	0.17
Ribosomal DNA	2	2	4.46	0.52	2	2	3.66	0.42	9	9	19.98	2.31	9	9	18.45	2.13	7	7	14.65	1.69
TOTAL	96	33	236.60	27.35	96	33	243.87	28.19	113	59	333.72	38.58	111	55	326.44	37.74	118	57	316.19	36.55

Number of clusters, genomic abundance and proportions of most abundant transposable elements classified according to Wicker et al. [1] in the five accessions of T. officinale apomictic lineage. Number of clusters showing high intra-family homogeneity are also indicated

The *T. officinale* genome is dominated by Class I LTR retrotransposons ranging from 21.4 % (185.2 Mb) to 31.5 % (272.4 Mb) depending on the accession. Ty1/*Copia* content was more constant among accessions (ranging from 9.8 to 11.7 %) than Ty3/*Gypsy* retroelements (9.0–17.7 %). Compared with Class I LTR retrotransposons, non-LTR retrotransposons and DNA transposons were relatively infrequent. Class II transposons only represented 1.2 % (10.7 Mb) to 4.8 % (41.1 Mb) of the genome. In all accessions, total read lengths of the top 10 clusters did not represent more than 8 % of the genome.

Abundance of ribosomal DNA was variable among accessions ranging from 0.4 % (3.7 Mb) to 2.3 % (20.0 Mb). Tandem repeats including satellites were not found in the most abundant clusters of all accessions representing a low-fraction of the repetitive compartment. Proportions of unclassified repeats account for 1.9–3.8 % of the genome and could include additional repeat types.

From histograms of pairwise sequence similarity between reads, we can infer aspects of relative age and dynamics of TEs. Overall in the clonal lineage, between 34.4 and 51.3 % of clusters showed evidence of recent TE proliferation as indicated by high similarity between reads. All classes and most superfamilies of TEs exhibited to some extent, proliferation of copies (Table 1). Other clusters displayed evidence for short and recent amplification, rapidly followed by divergence of copies as demonstrated by the largest number of clusters in model 5 (Additional file 3). Peaks of similarity scores were reached around 95.3–95.5 % for accessions 8 and 11 and at 95.7 % for accessions 3, 12 and 13 (Additional file 3).

### Genomic differences are driven mostly by few accession-specific repeat superfamilies

The abundance of the different repeat annotations, as estimated by read counts, differed between the five accessions at all levels of annotation (order, superfamily and family: likelihood ratio Chi-square test, *p* <0.0001, Additional file 4). The first clade including accessions 11 and 8, showed less overall TE content than the second clade comprising accessions 3, 12 and 13 (Fig. 1). This last clade mostly differed from the previous by showing a higher number of Ty3/*Gypsy Chromovirus* elements and to a lesser extent higher proportions of transposons and ribosomal DNA copies. This last clade was also more dynamic with 47.8–52.2 % (compared to 34.3 % in clade 1) of repeat showing evidence of ongoing TE proliferation (Table 1, Additional file 5).

Within the second clade (accessions 3, 12 and 13), differences observed at the superfamily level between accessions were due to a small number of specific TE copies. Accession 12 has more Ty3/*Gypsy Chromovirus* sequences (130.0 Mb accounting for 15.0 % of the genome) which were showing recent TE amplification (16 out of 36 clusters). In accessions 3 and 13, main differences were driven by DNA transposons increasing total repeat length (32.1–41.1 Mb compared to 23.2 Mb in accession 12, Table 1) and rate of ongoing proliferation (41.7–42.8 % of DNA transposon clusters compared to 37.5 % in accession 12, Table 1). Most dynamic annotated DNA transposons included *hAT*, *Mutator* and *Helitron* superfamilies (Additional file 5).

### Relationship between TE content and transcriptional activity

**Fig. 2:**
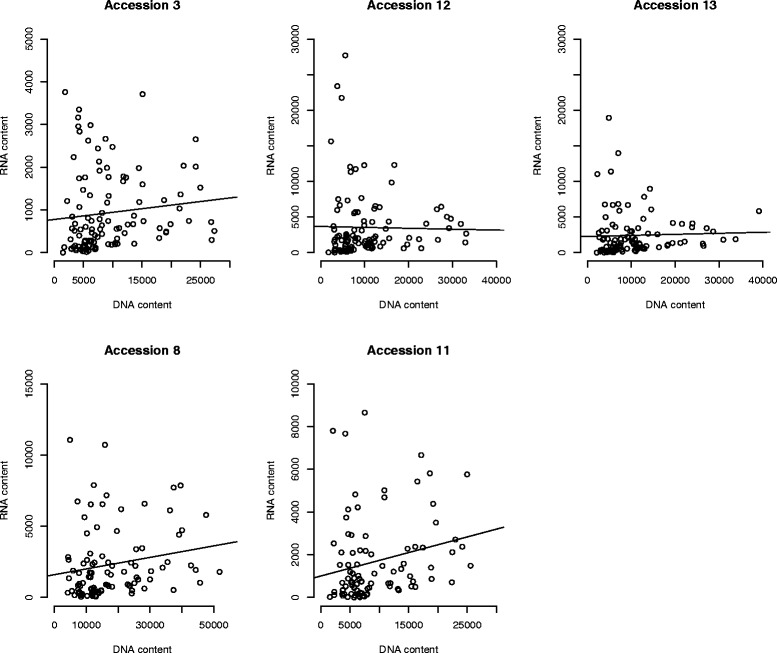
Transposable element genomic abundances in relation to transcription levels for all five accessions studied. DNA content on the x-axis and RNA content on the y-axis are expressed in number of reads per million at the cluster level. Expression levels (RNA content) are from [26]

To look at the relationship between TE genomic content and transcriptional activity, we used the newly made TE database to map previously generated RNA-Seq data [26]. Regression analyses showed weak but positive relationships between RNA and DNA content for several accessions which were statistically significant for accessions 8 and 11 (Fig. 2). Conversely, no relationship was observed in accessions 12 and 13. The absence of a positive trend in these two accessions is at least partially caused by a few TE families that are low-abundant at the DNA level but that are very highly transcribed (e.g. two LTR elements: *Copia Ale II* and *Copia Tork* and one DNA transposon: *Mutator*-like *Transposase* genes). However, TE families with highly homogeneous copies do not exhibit higher transcriptomic activity (Additional file 6).

Using reduced representation bisulfite sequencing, out of 623 filtered loci we identified 111 DMRs, that showed a range of variation from low to intermediate methylation or from intermediate to high methylation between accessions, and we detected 7 “true” DMRs that ranged from low to high methylation between accessions. From the 623 loci, 258 were annotated as functional transcripts and 159 as TEs. Among the detected DMRs, 48 were annotated as functional transcripts and 30 as TEs. A summary of annotated TEs that showed differential methylation levels is presented in Table 2. Most TEs exhibit low levels of transcriptional activity associated with high or moderate levels of methylation in leaf tissue. However, no clear correlation was observed between methylation levels and expression of TEs when comparing the five accessions. For instance, the LTR retrotransposon *Copia Ale II* and the DNA transposon *PIF-harbinger* are two highly transcribed elements showing various levels of transcription and methylation among the five apomict accessions (Table 2).

**Table 2:** Transposable elements exhibiting differences in CG methylation levels

TE sequence Annotation	Macranthoides 12			Macranthoides 13			Macranthoides 3			Macranthoides 11			Macranthoides 8		
	% CGm	DNA content	RNA content	% CGm	DNA content	RNA content	% CGm	DNA content	RNA content	% CGm	DNA content	RNA content	% CGm	DNA content	RNA content
LTR Copia AleII	0.18	19.26	751.75	0.30	20.64	36.82	0.14	22.93	392.60	0.09	72.44	205.34	0.23	48.78	228.26
LTR Copia Maximus/SIRE	0.85	93.54	0.00	0.88	41.26	0.00	0.95	80.21	0.00	0.04	159.25	0.00	1.00	317.67	0.00
DNA Transposon PIF-Harbinger	0.00	71.44	163.94	0.01	41.47	60.25	0.23	140.38	172.12	0.10	59.11	292.17	0.25	84.66	68.03
LTR Gypsy Ogre/Tat	0.71	349.49	17.76	0.77	#N/A	#N/A	0.74	337.44	8.87	0.67	403.89	0.00	0.69	808.12	0.00
DNA transposon hAT-Tip100	0.01	4.09	0.00	0.04	9.51	23.21	0.05	2.79	0.00	0.35	#N/A	#N/A	0.20	18.90	0.00
LTR Gypsy Ogre/Tat	0.98	349.49	17.76	0.85	#N/A	#N/A	0.15	337.44	8.87	0.00	403.89	0.00	0.00	808.12	0.00
LTR Copia Maximus/SIRE	0.00	37.91	0.00	0.42	39.14	0.00	0.02	30.32	0.00	0.00	45.45	0.00	0.00	45.62	0.00

Transposable elements showing differential levels of CG methylation among the five accessions studied. When possible, DNA and RNA contents were retrieved from the mapping analysis at the sequence level

## Discussion

This study is the first characterization of multiple repetitive compartments within a single asexual plant lineage. The genome of *T. officinale* is composed of highly heterogeneous and relatively low-abundant repeat families as shown previously in *T. kok saghyz* [46]. Transposable elements account for 27.4–38.6 % of the genomic content with high proportion of LTR retrotransposons (21.4–31.5 %). This repetitive compartment is typical of Angiosperms with most known plant TE families identified [1]. We characterized a high proportion of recent and transcribed TE families contributing to genetic diversity under asexuality. More precisely, we also identified accession-specific TE families exhibiting differences in transcription and methylation levels within the apomictic lineage. However, the variation in TE transcription was not clearly correlated to variation in methylation.

### The repetitive fraction of *T. officinale* is heterogeneous, young and dynamic

To survive, apomictic lineages need to control proliferation of most active TEs. A large part of TEs show peaks in sequence similarity scores between copies well below 100 %; as expected for TE families whose activities are under host genome control. In *T. officinale*, 50–70 % of the clusters are showing patterns of pairwise similarities that are consistent with relatively recent proliferation followed by rapid degeneration of copies. This specific pattern was observed previously in the genome of *Fritillaria* where copies had amplified simultaneously accompanied by low rates of deletion creating an ancient TE fraction that degenerated and diverged overtime [28].

On the other hand, over 1/3 to half of the TE families within the apomictic lineage appear to have highly conserved intra-family sequences reflecting their relatively young age and recent transposition activity. A previous study looking at a small number of retroelement sequences in four young asexual species including *T. officinale* came to the same conclusion [47]. Presence of highly conserved sequences can be used as a proxy to detect recent or still ongoing proliferation of TEs in the host genome as these TEs have not degenerated by mutation accumulation and have conserved the possibility of being active and inserted in a new location. In cotton, highly similar young LTR elements found only on chromosome 1 exhibit the same pattern and active transcription of these young LTRs generates siRNAs to maintain the silenced state of these TEs [35]. Due to such silencing, young and conserved *Gossypium* elements are not necessarily active and transposing. In the same way, *Taraxacum* TE families with highly homogeneous copies do not automatically exhibit higher transcriptional activity in leaf tissue. Activity and transposition of young TE could be restricted to earlier developmental stages and seed development; therefore, not detectable in the leaf transcriptome [48]. Still, in two of the analysed accessions, a few low-abundant TE families exhibit high transcriptomic levels. We can speculate that they may be escaping host genome control; however, even when these TEs also showed variable methylation between the accessions (see Table 2), there was no clear relation between methylation level and transcription level. Alternatively, transcription of repetitive sequences can be part of the silencing machinery targeting TEs via transcript degradation into small RNAs [35, 49].

### TE differences within one clonal lineage are governed by few repeat families

To characterize the potential actors of intra-clonal genomic divergence within one apomictic lineage, we compared five accessions of *T. officinale* genotype Macranthoides at the genomic, transcriptomic and epigenetic levels. We detected differences in TE genomic content but also in transcription and methylation levels which could have associated effects on nearby gene regulation and expression.

#### Differences in genomic content and proliferation rate

Differences in TE genomic content and proliferation rate were driven mostly by a few repeat families. Accession clustering based on TE content matched a previous clustering based on Single Nucleotide Polymorphisms (SNPs) and suggests the existence of two main clades [26]. The second clade differs from the first one, with higher overall TE content and more specifically, higher *Gypsy Chromovirus* Retrotransposon copy number. *Gypsy* intra-family sequences are highly conserved possibly reflecting a recent origin and incompletely or recently controlled by host genome. Within this second clade some accession-specific elements are showing higher genomic abundance coupled with highly conserved sequences. These elements are annotated as *Gypsy* LTR Retrotransposon Chromoviruses and some DNA transposons including *hAT*, *Mutator* and *Helitron*.

Retrotransposons seem to play a major role in driving genomic divergence in higher plants [50]. The few studies performed on intra-specific TE variability tend to show strikingly high variability in several Angiosperms. In *Helianthus*, an IRAP protocol was applied based on *Copia* and unknown LTR-elements: Wild accessions of *H. annuus* exhibit a number of polymorphic bands as high as among several *Helianthus* species suggesting continuous LTR retroelements activity after speciation [51]. Selfing also triggers large TE-related variability within species [52–54]. In *Aegilops speltoides*, fluctuations in TE copy number are lineage and TE-family specific [52]. Interestingly, in inbred lines of hybrid sunflower, TE genetic distance is more correlated to heterosis traits than SNP distance showing the possible phenotypic effects of TEs [54].

Special focus has been given to retrotransposon elements in Angiosperms as they usually contribute to large proportion of genomic coverage. However, despite their small size, DNA transposons can have also tremendous effect on plant genomes. Indeed, when transposing they can capture bit of host genome DNA sequence and profoundly affect its integrity [55]. In maize, *Helitrons* are responsible for creating thousands of new genomic insertions and unprecedented genic diversity between two inbred lines [56]. Other DNA transposons such as *hAT* and *PIF/Harbinger* have been reported in maize generating chromosomal rearrangements and gene duplications [57]. These effects coupled with a strong bottleneck mechanism could impact TE dynamics within and among populations [53, 58].

#### Specific TEs are showing variable transcriptional activity and differences in methylation levels

Besides changes in genomic abundance, identified TEs experiencing differential transcriptional activity accompanied by methylation level variation can reveal additional evidence for rapid diversification within an asexual lineage. Thus far, few but striking examples exist where studies combine both approaches in experimentally cloned organisms [59, 60]. These TEs have the potential to directly and indirectly affect gene regulation and expression by novel insertions, attracting methylation groups or producing small RNAs that can affect expression at other loci. Also, they are more prone to respond to environmental heterogeneity [19] possibly via epigenetic mechanisms.

Following hybridization and transition to asexuality, the epigenetic machinery including methylation levels can be altered. Apomict lineages offer a unique opportunity to investigate differences in methylation between accessions, their effects on TE release and potential impact on within-lineage divergence. Overall, we were able to detect 118 RRBS loci with variable methylation out of 623 loci (18.9 %) in CG context between the five accessions, providing evidence for methylation variation within one clonal lineage as previously shown in other *T. officinale* lineages [61]. In the context of within-lineage divergence, transcriptional variation in TEs and TE-related silencing genes are also observed accompanied by heritable variation in functionally relevant pathways [26, 61]. Interestingly, 25.4 and 40.7 % of the detected DMRs are annotated as TEs or functional gene transcripts, respectively. Differentially methylated TEs are potentially involved in functional differentiation and early genomic divergence between accessions of the same apomictic lineage. For instance, two conserved TE copies exhibiting variable levels of expression and non-consistent methylation variation were detected. *Copia AleII* is a LTR-retrotransposon present in low abundance in some plant genomes and not well characterized. Conversely, *PIF-Harbinger* is an autonomous DNA transposon superfamily identified in several plant genomes and responsible for the generation and amplification of miniature inverted repeat TEs (MITEs). Tourist-like and Stowaway elements (two predominant MITE families) appear to play a significant role in gene and genome evolution as they are abundant and insert preferentially into genes [62, 63]. More work is needed to assess the direct regulation of these TEs (and nearby genes) by methylation.

## Conclusion

We reveal highly heterogeneous, young and dynamic TE families contributing to transcriptomic and genomic divergence within one apomictic lineage of *T. officinale*. Our results show that recent and active TEs trigger genetic variation, which we speculate could be particularly relevant for asexual lineages facing environmental heterogeneity. Additionally, we report specific TE families whose differential transcription between accessions is accompanied by variable methylation levels within one single apomictic lineage, although there was not a simple correlation between expression and methylation levels. These elements may be more prone to affect new regulatory pathways and/or affect nearby gene expression. Further studies of these peculiar TEs will shed light on the direct effects of TE transposition on functional genes and pathways. Remobilized TEs may be important for microevolution processes and allow species with limited sexual recombination to be more plastic and adapt to changing environments.

## Additional files

Additional file 1: Geographical origin of the biological material. (DOCX 12 kb)
Additional file 2: Histogram of methylation level occurrences in CG context. (PNG 7 kb)
Additional file 3: Intra-family heterogeneity of most abundant TE families in the genomes of the five accessions of T. officinale. Number of clusters are presented for each model described in the method section. (XLSX 9 kb)
Additional file 4: Statistical analyses of the relative abundance of TE classes between accessions, as estimated by read counts, at three hierarchical levels of annotation (Likelihood Ratio Chi-square test). (XLSX 9 kb)
Additional file 5: Number of clusters, genomic abundance and proportions of most abundant transposable elements classified according to Wicker et al. [1] in the five accessions of T. officinale apomictic lineage. (XLSX 13 kb)
Additional file 6: Transposable element genomic abundance in relation with transcriptomic level and read similarity, for all five accessions studied. Each TE cluster is marked as proliferating (green circle) or not (yellow diamond) following method explained in main text. DNA content on the x-axis and RNA content on the y-axis are expressed in number of reads per million at the cluster level. (JPG 62 kb)

## Abbreviations

BIC: Bayesian information criterion
BLAST: Basic local alignment search tool
DMP: Differentially methylated position
DMR: Differentially methylated region
DNA: Desoxyribonucleic acid
FPKM: Fragments per kilobase of exon per million
IRAP: Inter-retrotransposon amplified polymorphism
LTR: Long terminal repeat
MITE: Miniature inverted transposable element
RNA: Ribonucleic acid
RRBS: Reduced representation bisulfite sequencing
siRNA: Small interfering ribonucleic acid
SNP: Single nucleotide polymorphism
TE: Transposable element
