# Supplementary material for: Recent and dynamic transposable elements contribute to genomic divergence under asexuality
Source: BMC Genomics. 2016 Nov 7;17:884. doi: 10.1186/s12864-016-3234-9 (PMC5100183; doi:10.1186/s12864-016-3234-9)
Supplement: Additional file 1: — Geographical origin of the biological material. (DOCX 12 kb) [file 12864_2016_3234_MOESM1_ESM.docx]

| Acc. | Sample Name | Country of origin | Coordinates |
| --- | --- | --- | --- |
| 11 | Macra11 | Germany | 50° 53' 48'' N 13° 50' 03'' E |
| 12 | Macra 1280-S1 | Czech republic | 49° 20' 56'' N 17° 18' 31'' E |
| 13 | Macra 1280-S2 | Czech republic | 49° 20' 56'' N 17° 18' 31'' E |
| 3 | Macra3 | Germany | 50° 47' 15'' N 13° 51' 22'' E |
| 8 | Macra8 | Germany | 50° 53' 48'' N 13° 50' 03'' E |
