# Supplementary figures and images for: Recent and dynamic transposable elements contribute to genomic divergence under asexuality
Source: BMC Genomics. 2016 Nov 7;17:884. doi: 10.1186/s12864-016-3234-9 (PMC5100183; doi:10.1186/s12864-016-3234-9)

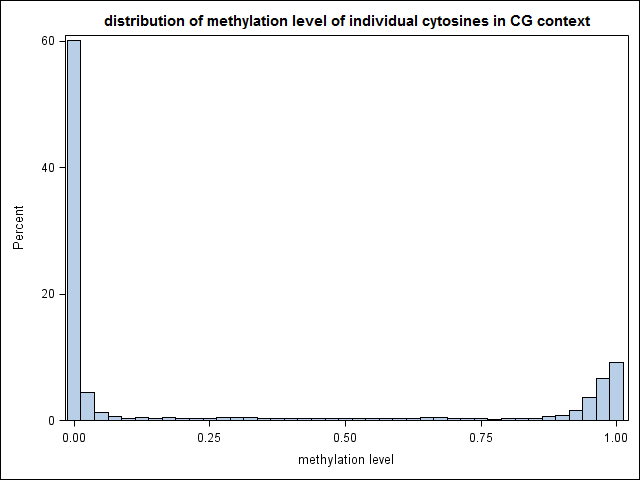

Supplement: Additional file 2: — Histogram of methylation level occurrences in CG context. (PNG 7 kb) [file 12864_2016_3234_MOESM2_ESM.png]

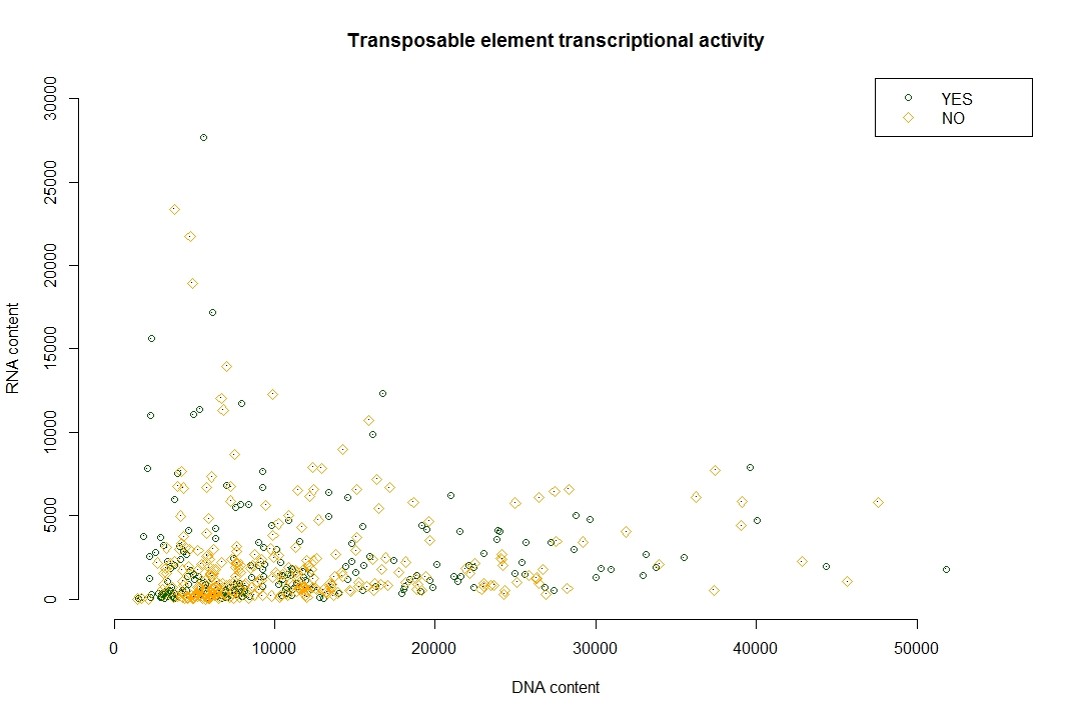

Supplement: Additional file 6: — Transposable element genomic abundance in relation with transcriptomic level and read similarity, for all five accessions studied. Each TE cluster is marked as proliferating (green circle) or not (yellow diamond) following method explained in main text. DNA content on the x-axis and RNA content on the y-axis are expressed in number of reads per million at the cluster level. (JPG 62 kb) [file 12864_2016_3234_MOESM6_ESM.jpg]
